# 2-Hy­droxy-5-{[(*E*)-4-meth­oxy­benzyl­idene]aza­nium­yl}benzoate

**DOI:** 10.1107/S1600536810036172

**Published:** 2010-09-11

**Authors:** M. Nawaz Tahir, Muhammad Ilyas Tariq, Shahbaz Ahmad, Muhammad Sarfraz

**Affiliations:** a Department of Physics, University of Sargodha, Sargodha, Pakistan; b Department of Chemistry, University of Sargodha, Sargodha, Pakistan

## Abstract

In the title zwitterion, C_15_H_13_NO_4_, obtained from the condensation of 5-amino­salicylic acid and 4-meth­oxy­benz­alde­hyde, the 4-hydoxyanilinic group of the 5-amino­salicylic acid moiety and the 4-meth­oxy­benzaldehyde moiety are twisted with respect to one another, making a dihedral angle of 10.37 (7)°. The carboxyl­ate group makes a dihedral angle of 5.7 (2)° with the parent 4-hydoxyanilinic group. An intra­molecular O—H⋯O hydrogen bond forms an *S*(6) ring motif. In the crystal, inter­molecular C—H⋯O and N—H⋯O hydrogen bonds with *R*_2_^1^(7) ring motifs link the mol­ecules into infinite chains extending along the *c* axis. The occurence of slipped π–π stacking between symmetry-related aromatic rings reinforces the packing.

## Related literature

For the related structures, see: Ashiq *et al.* (2010 ▶); Bryan *et al.* (1978 ▶). For graph-set notation, see: Bernstein *et al.* (1995 ▶). For π–π stacking, see: Janiak (2000 ▶).
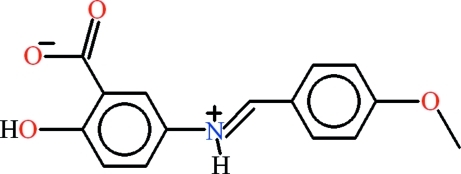


## Experimental

### 

#### Crystal data

C_15_H_13_NO_4_
*M* *_r_* = 271.26
Monoclinic, 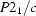

*a* = 13.4369 (12) Å
*b* = 8.5619 (8) Å
*c* = 12.6653 (11) Å
β = 118.004 (3)°
*V* = 1286.5 (2) Å^3^
*Z* = 4
Mo *K*α radiation
μ = 0.10 mm^−1^
*T* = 296 K
0.26 × 0.20 × 0.18 mm

#### Data collection

Bruker Kappa APEXII CCD diffractometer
Absorption correction: multi-scan (*SADABS*; Bruker, 2005 ▶) *T*_min_ = 0.982, *T*_max_ = 0.987
9561 measured reflections
2320 independent reflections
1583 reflections with *I* > 2σ(*I*)
*R*_int_ = 0.031

#### Refinement

*R*[*F* ^2^ > 2σ(*F* ^2^)] = 0.050
*wR*(*F* ^2^) = 0.143
*S* = 1.02
2320 reflections
182 parameters
H-atom parameters constrained
Δρ_max_ = 0.28 e Å^−3^
Δρ_min_ = −0.18 e Å^−3^

### 

Data collection: *APEX2* (Bruker, 2009 ▶); cell refinement: *SAINT* (Bruker, 2009 ▶); data reduction: *SAINT*; program(s) used to solve structure: *SHELXS97* (Sheldrick, 2008 ▶); program(s) used to refine structure: *SHELXL97* (Sheldrick, 2008 ▶); molecular graphics: *ORTEP-3 for Windows* (Farrugia, 1997 ▶) and *PLATON* (Spek, 2009 ▶); software used to prepare material for publication: *WinGX* (Farrugia, 1999 ▶) and *PLATON*.

## Supplementary Material

Crystal structure: contains datablocks global, I. DOI: 10.1107/S1600536810036172/dn2599sup1.cif

Structure factors: contains datablocks I. DOI: 10.1107/S1600536810036172/dn2599Isup2.hkl

Additional supplementary materials:  crystallographic information; 3D view; checkCIF report

## Figures and Tables

**Table 1:** Hydrogen-bond geometry (Å, °)

*D*—H⋯*A*	*D*—H	H⋯*A*	*D*⋯*A*	*D*—H⋯*A*
O3—H3⋯O1	0.90	1.62	2.4816 (19)	159
N1—H1⋯O2^i^	0.86	1.90	2.7408 (19)	166
C8—H8⋯O1^ii^	0.93	2.47	3.179 (2)	133
C14—H14⋯O2^i^	0.93	2.30	3.183 (2)	159
C15—H15*A*⋯O3^iii^	0.96	2.56	3.393 (3)	145

Symmetry codes: (i) 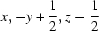; (ii) 
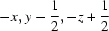
; (iii) 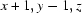.

**Table 2:** π–π stacking inter­actions (Å, °) *Cg*1 and *Cg*2 are the centroids of the C2–C7 and C9–C14 rings, respectively.

*Cg*⋯*Cg*	centroid–centroid distance	mean inter­planar distance^*a*^	slippage angle^*b*^
*Cg*1⋯*Cg*1^i^	3.7848 (13)	3.428 (1)	25.1
*Cg*1⋯*Cg*2^ii^	3.8456 (13)	3.567 (1)	22.0

Symmetry codes: (i) −*x*, 1 − *y*, −*z*, (ii) −*x*, −*y*, −*z*. Notes: (*a*) distance from one plane to the neighbouring centroid; (*b*) angle subtended by the inter­centroid vector to the plane normal. For details, see: Janiak (2000 ▶).

**Fig. 1.**
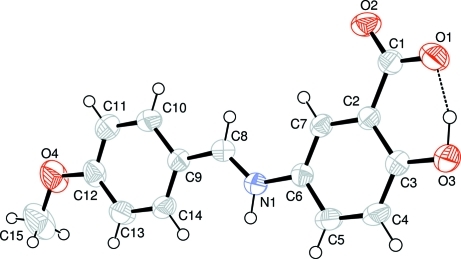
View of the title compound with the atom numbering scheme. The thermal ellipsoids are drawn at the 50% probability level. H-atoms are shown by small circles of arbitrary radii.

**Fig. 2.**
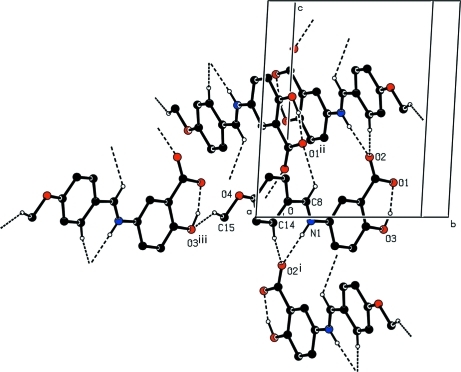
Partial packing view showing the hydrogen bonding pattern. Hydrogen bonds are shown as dashed lines. H atoms not involved in hydrogen bondngs have been omitted for clarity. [Symmetry codes: (i) x, -y+1/2, z-1/2; (ii) -x, y-1/2, -z+1/2; (iii) x+1, y-1, z].

## supplementary crystallographic information

### Comment

The title compound (I) has been synthesized as a potential ligand which could be used with various metals.

The title compound (I) is a Zwitterion. In (I), the group A (C2—C7/N1/O3) of 5-aminosalicylic acid moiety and the 4-methoxybenzaldehyde moiety B (C8—C15/O4) are planar with r. m. s. deviations of 0.0076 and 0.0255 Å, respectively. The dihedral angle between A/B is 10.37 (7)°. The carboxylate group C (O1/C1/O2) is oriented at a dihedral angle of 5.73 (24)° with the parent group A (Fig. 1). The title molecule is closely related to *p*-[(*p*-methoxybenzylidene)amino]phenol (Bryan *et al.*, 1978) and *N*-benzyl-*N'*-{6- [(4-carboxylatobenzyl)aminocarbonyl]-2-pyridylmethyl}guanidinium (Ashiq *et al.*, 2010)

The values of the C═O bond [1.241 (2), 1.269 (3) Å] are in agreement with the value, 1.235 (3)–1.261 (3) Å, reported for the *N*-benzyl-*N'*-{6- [(4-carboxylatobenzyl)aminocarbonyl]-2-pyridylmethyl}guanidinium (Ashiq *et al.*, 2010). In the title compound S(6) ring motifs (Bernstein *et al.*, 1995), is formed due to intramolecular H-bondings of O—H···O type (Table 1, Fig. 2). There exist *R*_2_^1^(7) ring motif due to intermolecular H-bondings of C—H···O and N—H···O types linking the molecules to form infinite one dimensional chains extending along the crystallographic *c*-axis.

The occurence of slippest π–π stacking (Janiak, 2000) between symmetry related aromatic rings play an important role in stabilizing the packing.

### Experimental

Equimolar quantities of 5-aminosalicylic acid and 4-anisaldehyde were refluxed in methanol for 30 min resulting in clear brown solution. The solution was kept at room temperature which affoarded light brown prisms after 72 h.

### Refinement

All H atoms attached to C atoms and N atom were fixed geometrically and treated as riding with C—H = 0.95 Å (methyl) or 0.93 Å (aromatic) and N—H = 0.86 Å with U_iso_(H) = 1.2U_eq_(C_aromatic_ or N) or U_iso_(H) = 1.5U_eq_(C_methyl_). H atoms of the hydroxyl group was located in difference Fourier maps and included in the subsequent refinement using restraints (O—H = 0.85 (1) Å) with U_iso_(H) = 1.5U_eq_(O). In the last cycles of refinement this hydrogen was treated as riding on its parent O atom.

### Crystal data

**Table:**

C_15_H_13_NO_4_	*F*(000) = 568
*M**_r_* = 271.26	*D*_x_ = 1.401 Mg m^−^^3^
Monoclinic, *P*2_1_/*c*	Mo *K*α radiation, λ = 0.71073 Å
Hall symbol: -P 2ybc	Cell parameters from 1583 reflections
*a* = 13.4369 (12) Å	θ = 2.9–25.3°
*b* = 8.5619 (8) Å	µ = 0.10 mm^−^^1^
*c* = 12.6653 (11) Å	*T* = 296 K
β = 118.004 (3)°	Prism, light brown
*V* = 1286.5 (2) Å^3^	0.26 × 0.20 × 0.18 mm
*Z* = 4	

### Data collection

**Table:**

Bruker Kappa APEXII CCD diffractometer	2320 independent reflections
Radiation source: fine-focus sealed tube	1583 reflections with *I* > 2σ(*I*)
graphite	*R*_int_ = 0.031
Detector resolution: 8.10 pixels mm^-1^	θ_max_ = 25.3°, θ_min_ = 2.9°
ω scans	*h* = −15→16
Absorption correction: multi-scan (*SADABS*; Bruker, 2005)	*k* = −10→10
*T*_min_ = 0.982, *T*_max_ = 0.987	*l* = −15→10
9561 measured reflections	

### Refinement

**Table:**

Refinement on *F*^2^	Primary atom site location: structure-invariant direct methods
Least-squares matrix: full	Secondary atom site location: difference Fourier map
*R*[*F*^2^ > 2σ(*F*^2^)] = 0.050	Hydrogen site location: inferred from neighbouring sites
*wR*(*F*^2^) = 0.143	H-atom parameters constrained
*S* = 1.02	*w* = 1/[σ^2^(*F*_o_^2^) + (0.0831*P*)^2^ + 0.1187*P*] where *P* = (*F*_o_^2^ + 2*F*_c_^2^)/3
2320 reflections	(Δ/σ)_max_ < 0.001
182 parameters	Δρ_max_ = 0.28 e Å^−^^3^
0 restraints	Δρ_min_ = −0.18 e Å^−^^3^

### Special details

**Table:**

Geometry. All esds (except the esd in the dihedral angle between two l.s. planes) are estimated using the full covariance matrix. The cell esds are taken into account individually in the estimation of esds in distances, angles and torsion angles; correlations between esds in cell parameters are only used when they are defined by crystal symmetry. An approximate (isotropic) treatment of cell esds is used for estimating esds involving l.s. planes.
Refinement. Refinement of *F*^2^ against ALL reflections. The weighted *R*-factor *wR* and goodness of fit *S* are based on *F*^2^, conventional *R*-factors *R* are based on *F*, with *F* set to zero for negative *F*^2^. The threshold expression of *F*^2^ > σ(*F*^2^) is used only for calculating *R*-factors(gt) *etc*. and is not relevant to the choice of reflections for refinement. *R*-factors based on *F*^2^ are statistically about twice as large as those based on *F*, and *R*- factors based on ALL data will be even larger.

### Fractional atomic coordinates and isotropic or equivalent isotropic displacement parameters (Å^2^)

**Table:**

	*x*	*y*	*z*	*U*_iso_*/*U*_eq_	
O1	−0.19046 (12)	0.62390 (19)	0.15961 (12)	0.0667 (5)	
O2	−0.03306 (11)	0.49725 (16)	0.27753 (10)	0.0510 (4)	
O3	−0.29868 (11)	0.56256 (18)	−0.05677 (12)	0.0613 (5)	
H3	−0.2728	0.6015	0.0176	0.092*	
O4	0.50744 (13)	−0.1897 (2)	0.09919 (15)	0.0857 (6)	
N1	0.04782 (11)	0.17725 (17)	−0.01733 (13)	0.0405 (4)	
H1	0.0329	0.1255	−0.0813	0.049*	
C1	−0.11320 (15)	0.5260 (2)	0.17761 (15)	0.0406 (5)	
C2	−0.11993 (13)	0.4447 (2)	0.06936 (14)	0.0353 (4)	
C3	−0.21322 (15)	0.4691 (2)	−0.04323 (16)	0.0437 (5)	
C4	−0.21754 (16)	0.3965 (3)	−0.14312 (17)	0.0548 (6)	
H4	−0.2793	0.4128	−0.2178	0.066*	
C5	−0.13155 (15)	0.3009 (2)	−0.13273 (16)	0.0507 (5)	
H5	−0.1350	0.2530	−0.2002	0.061*	
C6	−0.03929 (14)	0.2756 (2)	−0.02139 (15)	0.0382 (4)	
C7	−0.03399 (13)	0.3462 (2)	0.07921 (15)	0.0371 (4)	
H7	0.0273	0.3276	0.1537	0.044*	
C8	0.14683 (14)	0.1574 (2)	0.07203 (16)	0.0421 (5)	
H8	0.1627	0.2105	0.1423	0.051*	
C9	0.23438 (14)	0.0614 (2)	0.07311 (16)	0.0413 (5)	
C10	0.33683 (16)	0.0561 (3)	0.17828 (17)	0.0548 (6)	
H10	0.3447	0.1116	0.2449	0.066*	
C11	0.42584 (16)	−0.0289 (3)	0.18534 (18)	0.0633 (7)	
H11	0.4934	−0.0315	0.2562	0.076*	
C12	0.41467 (16)	−0.1115 (3)	0.08600 (18)	0.0547 (6)	
C13	0.31336 (17)	−0.1093 (3)	−0.01877 (18)	0.0541 (5)	
H13	0.3056	−0.1665	−0.0846	0.065*	
C14	0.22442 (16)	−0.0231 (2)	−0.02569 (17)	0.0476 (5)	
H14	0.1570	−0.0209	−0.0967	0.057*	
C15	0.5007 (2)	−0.2741 (4)	−0.0010 (3)	0.1025 (10)	
H15A	0.5716	−0.3243	0.0206	0.154*	
H15B	0.4836	−0.2032	−0.0660	0.154*	
H15C	0.4424	−0.3515	−0.0250	0.154*	

### Atomic displacement parameters (Å^2^)

**Table:**

	*U*^11^	*U*^22^	*U*^33^	*U*^12^	*U*^13^	*U*^23^
O1	0.0623 (9)	0.0904 (12)	0.0448 (8)	0.0283 (9)	0.0230 (7)	−0.0037 (8)
O2	0.0533 (8)	0.0604 (9)	0.0315 (7)	0.0028 (7)	0.0135 (6)	0.0023 (6)
O3	0.0447 (8)	0.0827 (11)	0.0461 (8)	0.0220 (7)	0.0127 (7)	−0.0035 (7)
O4	0.0628 (10)	0.1237 (15)	0.0748 (11)	0.0473 (10)	0.0358 (9)	0.0216 (11)
N1	0.0403 (8)	0.0448 (9)	0.0360 (8)	0.0020 (7)	0.0176 (7)	−0.0029 (7)
C1	0.0401 (10)	0.0465 (11)	0.0365 (10)	−0.0018 (9)	0.0189 (9)	0.0033 (8)
C2	0.0337 (9)	0.0390 (10)	0.0332 (9)	−0.0022 (8)	0.0157 (8)	0.0026 (8)
C3	0.0361 (9)	0.0522 (12)	0.0392 (10)	0.0055 (9)	0.0147 (8)	0.0013 (9)
C4	0.0436 (11)	0.0728 (14)	0.0321 (10)	0.0118 (11)	0.0046 (9)	−0.0036 (10)
C5	0.0472 (11)	0.0620 (13)	0.0345 (10)	0.0066 (10)	0.0120 (9)	−0.0085 (9)
C6	0.0360 (9)	0.0402 (10)	0.0388 (10)	0.0019 (8)	0.0177 (8)	0.0009 (8)
C7	0.0320 (9)	0.0417 (10)	0.0332 (9)	−0.0005 (8)	0.0118 (8)	0.0050 (8)
C8	0.0408 (10)	0.0478 (11)	0.0362 (10)	−0.0014 (9)	0.0168 (8)	−0.0032 (8)
C9	0.0369 (10)	0.0465 (11)	0.0385 (10)	0.0012 (8)	0.0161 (8)	0.0009 (8)
C10	0.0445 (11)	0.0755 (15)	0.0372 (11)	0.0052 (10)	0.0131 (9)	−0.0055 (10)
C11	0.0399 (11)	0.0946 (18)	0.0445 (12)	0.0129 (11)	0.0108 (10)	0.0063 (12)
C12	0.0457 (11)	0.0698 (14)	0.0526 (13)	0.0199 (11)	0.0264 (10)	0.0174 (11)
C13	0.0569 (12)	0.0628 (13)	0.0435 (11)	0.0120 (11)	0.0243 (10)	0.0011 (10)
C14	0.0393 (10)	0.0557 (12)	0.0399 (10)	0.0071 (9)	0.0120 (9)	−0.0008 (9)
C15	0.103 (2)	0.123 (2)	0.107 (2)	0.0604 (19)	0.0700 (18)	0.0201 (19)

### Geometric parameters (Å, °)

**Table:**

O1—C1	1.269 (2)	C6—C7	1.381 (2)
O2—C1	1.242 (2)	C7—H7	0.9300
O3—C3	1.343 (2)	C8—C9	1.430 (2)
O3—H3	0.9015	C8—H8	0.9300
O4—C12	1.354 (2)	C9—C10	1.396 (2)
O4—C15	1.426 (3)	C9—C14	1.396 (3)
N1—C8	1.291 (2)	C10—C11	1.367 (3)
N1—C6	1.423 (2)	C10—H10	0.9300
N1—H1	0.8600	C11—C12	1.389 (3)
C1—C2	1.502 (2)	C11—H11	0.9300
C2—C7	1.388 (2)	C12—C13	1.385 (3)
C2—C3	1.404 (2)	C13—C14	1.372 (3)
C3—C4	1.386 (3)	C13—H13	0.9300
C4—C5	1.371 (3)	C14—H14	0.9300
C4—H4	0.9300	C15—H15A	0.9600
C5—C6	1.390 (2)	C15—H15B	0.9600
C5—H5	0.9300	C15—H15C	0.9600
			
C3—O3—H3	101.5	N1—C8—H8	117.0
C12—O4—C15	118.07 (18)	C9—C8—H8	117.0
C8—N1—C6	126.96 (16)	C10—C9—C14	118.36 (17)
C8—N1—H1	116.5	C10—C9—C8	117.77 (17)
C6—N1—H1	116.5	C14—C9—C8	123.85 (16)
O2—C1—O1	123.99 (17)	C11—C10—C9	121.33 (19)
O2—C1—C2	119.34 (16)	C11—C10—H10	119.3
O1—C1—C2	116.65 (15)	C9—C10—H10	119.3
C7—C2—C3	119.37 (16)	C10—C11—C12	119.54 (18)
C7—C2—C1	120.59 (15)	C10—C11—H11	120.2
C3—C2—C1	120.04 (15)	C12—C11—H11	120.2
O3—C3—C4	118.93 (16)	O4—C12—C13	124.0 (2)
O3—C3—C2	121.38 (16)	O4—C12—C11	115.99 (18)
C4—C3—C2	119.68 (16)	C13—C12—C11	120.05 (18)
C5—C4—C3	120.52 (17)	C14—C13—C12	120.22 (19)
C5—C4—H4	119.7	C14—C13—H13	119.9
C3—C4—H4	119.7	C12—C13—H13	119.9
C4—C5—C6	120.05 (17)	C13—C14—C9	120.49 (17)
C4—C5—H5	120.0	C13—C14—H14	119.8
C6—C5—H5	120.0	C9—C14—H14	119.8
C7—C6—C5	120.18 (16)	O4—C15—H15A	109.5
C7—C6—N1	122.70 (15)	O4—C15—H15B	109.5
C5—C6—N1	117.12 (16)	H15A—C15—H15B	109.5
C6—C7—C2	120.18 (15)	O4—C15—H15C	109.5
C6—C7—H7	119.9	H15A—C15—H15C	109.5
C2—C7—H7	119.9	H15B—C15—H15C	109.5
N1—C8—C9	126.07 (17)		

### Hydrogen-bond geometry (Å, °)

**Table:**

*D*—H···*A*	*D*—H	H···*A*	*D*···*A*	*D*—H···*A*
O3—H3···O1	0.90	1.62	2.4816 (19)	159
N1—H1···O2^i^	0.86	1.90	2.7408 (19)	166
C8—H8···O1^ii^	0.93	2.47	3.179 (2)	133
C14—H14···O2^i^	0.93	2.30	3.183 (2)	159
C15—H15A···O3^iii^	0.96	2.56	3.393 (3)	145

Symmetry codes: (i) *x*, −*y*+1/2, *z*−1/2; (ii) −*x*, *y*−1/2, −*z*+1/2; (iii) *x*+1, *y*−1, *z*.

### Table 2 π–π stacking interactions (Å, °)

Cg1 and Cg2 are the centroids of the C2–C7 and C9–C14 rings, respectively.

**Table:**

Cg···Cg	centroid–centroid distance	mean interplanar distance^a^	slippage angle^b^
Cg1···Cg1^i^	3.7848 (13)	3.428 (1)	25.1
Cg1···Cg2^ii^	3.8456 (13)	3.567 (1)	22.0

Symmetry codes: (i) -x, 1-y, -z, (ii) -x, -y, -z. Notes: (a) distance from one plane to the neighbouring centroid; (b) angle subtended by the intercentroid vector to the plane normal. For details, see: Janiak (2000).
